# Anhedonia and anxiety underlying depressive symptomatology have distinct effects on reward-based decision-making

**DOI:** 10.1371/journal.pone.0186473

**Published:** 2017-10-23

**Authors:** Katia M. Harlé, Dalin Guo, Shunan Zhang, Martin P. Paulus, Angela J. Yu

**Affiliations:** 1 Department of Psychiatry, UCSD, La Jolla, CA, United States of America; 2 Department of Electrical and Computer Engineering, UCSD, La Jolla, CA, United States of America; 3 Department of Cognitive Science, UCSD, La Jolla, CA, United States of America; 4 Laureate Institute for Brain Research, Tulsa, Oklahoma, United States of America; University of Sheffield, UNITED KINGDOM

## Abstract

Depressive pathology, which includes both heightened negative affect (e.g., anxiety) and reduced positive affect (e.g., anhedonia), is known to be associated with sub-optimal decision-making, particularly in uncertain environments. Here, we use a computational approach to quantify and disambiguate how individual differences in these affective measures specifically relate to different aspects of learning and decision-making in reward-based choice behavior. Fifty-three individuals with a range of depressed mood completed a two-armed bandit task, in which they choose between two arms with fixed but unknown reward rates. The decision-making component, which chooses among options based on current expectations about reward rates, is modeled by two different decision policies: a learning-independent Win-stay/Lose-shift (WSLS) policy that ignores all previous experiences except the last trial, and Softmax, which prefers the arm with the higher expected reward. To model the learning component for the Softmax choice policy, we use a Bayesian inference model, which updates estimated reward rates based on the observed history of trial outcomes. Softmax with Bayesian learning better fits the behavior of 55% of the participants, while the others are better fit by a learning-independent WSLS strategy. Among Softmax “users”, those with higher anhedonia are less likely to choose the option estimated to be most rewarding. Moreover, the Softmax parameter mediates the inverse relationship between anhedonia and overall monetary gains. On the other hand, among WSLS “users”, higher state anxiety correlates with increasingly better ability of WSLS, relative to Softmax, to explain subjects’ trial-by-trial choices. In summary, there is significant variability among individuals in their reward-based, exploratory decision-making, and this variability is at least partly mediated in a very specific manner by affective attributes, such as hedonic tone and state anxiety.

## Introduction

Effective reward-seeking behavior and decision-making typically require balancing actions already known to have positive outcomes and exploration of new, less-travelled paths to get potentially even better results. In real life, it is in such complex, uncertain environments that individuals with depressed mood make sub-optimal decisions. Moreover, while depressed mood is known to alter reward-based decision-making, the evidence is somewhat mixed and suggests underlying complexity [1]. One possible source of complexity is the multi-dimensional nature of this psychological state, with deficits in both negative emotionality (e.g., elevated guilt, anxiety) and positive affect (decreased happiness, pleasure anticipation). Such complexity is likely to account for a wide range depressive profiles and substantial individual differences in their underlying cognitive alterations (e.g. type of processing deficit). A precise quantification of how these distinct emotional attributes may differentially impact reward processing is therefore important for both basic cognitive science and clinical research, as it may improve the understanding, detection, classification, and treatment of many psychiatric conditions with known deficits in reward-based decision-making.

In depressed individuals, anhedonia, i.e., reduced capacity to experience and anticipate pleasure from life experiences, is associated with reduced positive affect ratings and weaker neural responsiveness to rewarding stimuli such as positive social cues and money [2, 3]. It has been noted that depressed individuals’ behavior is less modulated by reinforcement history in reward learning paradigms [4]. Such reward hyposensitivity has been linked to attenuated recruitment of reward-processing neural areas (e.g., ventral striatum, nucleus accumbens) and reduced fronto-striatal connectivity [5, 6]. In contrast, negative affective states common in depression, such as anxiety, appear to primarily increase sensitivity to and expectations of negative outcomes, including loss, punishments, and errors [7, 8]. Together, these findings suggest that, within depressed mood, low hedonic tone may affect reward-seeking behavior by altering reward sensitivity in choice behavior, or the learning of reward rates under uncertainty, whereas high anxiety may specifically affect reaction to negative outcomes while having less prominent overall effects on reward learning or decision-making.

Given the overlap of these cognitive functions in complex behavioral scenarios, we adopt a mathematically precise Bayesian modeling framework to investigate learning and decision-making behavior in individuals with a range of depressive severity, while they perform a binary-choice version [9] of a classic exploratory decision-making paradigm known as a multi-armed bandit task [10, 11]. Thus, in this study, we specifically focus on reward processing and reward-based decision-making, as in traditional bandit paradigms with win or no-win outcomes, rather than punishment or loss based decision-making. We previously suggested that two separable computational components underlie human choice behavior in the bandit task [12, 13]: a *learning component*, whereby one updates internal beliefs about unknowns in the environment based on successive observations of outcomes, and a *decision-making component*, whereby one selects an action based on those beliefs. Specifically, we quantify the learning and decision-making processes by using a Bayesian ideal observer model assuming decision-makers continuously update their beliefs of the environment based on each new observation (Dynamic Belief Model/DBM; [14]). DBM assumes that the observer assumes environmental statistics to undergo discrete, unsignaled change-points at a typical timescale (captured by a stability parameter), and therefore updates one’s beliefs about the environment (e.g. reward rate) by exponentially forgetting past observations, as well as continually injecting a fixed prior belief about the environment (since the hidden state is assumed to be re-drawn from a fixed prior distribution whenever a change-point occurs) [14]. We note that the stability parameter γ in the DBM controls the rate of exponential forgetting, which is related but not mathematically equivalent to the standard RL learning rate (14). Even though the true reward rates in our task stay fixed throughout each game, we use DBM to model human learning due to our prior findings that this is how subjects behave in a variety of behavioral tasks (12, 13, 14).

To model decision-making, we use two well-known and psychologically meaningful decision policies from the reinforcement learning (RL) literature: Win-Stay/Lose-Shift (WSLS)[9] and Softmax[15, 16]. While WSLS is heuristic model, which does not rely on Bayesian (or any other type of) learning, Softmax is coupled with DBM. Given findings reviewed above, and our specific focus on reward-based decision-making (vs penalty-based decisions), we hypothesize that individuals with higher anhedonia may specifically exhibit reduced reward sensitivity, which should be reflected in the computational processes underlying reward processing/learning and associated decision-making, i.e., in their decision policy parameters. In contrast, we did not expect any significant or strong relationship between decision policy parameters of reward sensitivity and negative affect measures, such as state anxiety, or overall depression severity. That is, depressed individuals with primarily heighted negative affect should not show altered computational parameters of reward sensitivity. This is because, as reviewed above, negative emotionality may more specifically modulate punishment processing and loss avoidance (not assessed here) rather than reward-based decision-making.

## Materials and methods

### Participants and procedures

Fifty-three undergraduate students (71% female; mean age = 20.5, age range:18–26) participated in this study, which was approved by the UCSD Human Research Protections Program. They signed up through the online UCSD SONA system, and then completed phone-screening and an on-line Beck Depression Inventory (BDI-II; [17]). We recruited a target of 25% (1 our of 4) of participants with no significant depression level (i.e., BDI-II score < 8), while the remaining part of recruited participants were included on the basis of minimal depression severity (i.e., BDI-II score > 8). Other inclusion criteria included a) being in good general health on the basis of brief review of medical history, and b) sufficient proficiency in English to understand and complete all study procedures. Exclusion criteria included: lifetime history of psychotic, bipolar or obsessive-compulsive disorder, history of current alcohol or substance dependence, recent history of (i.e., within last 6 months) or currently taking any antidepressant or psychotropic medications (except occasional sleep aid). Qualified subjects completed the experiment in the laboratory, which included a brief set of questionnaires and the Bandit Task. Participants completed the State Anxiety Inventory (STAI; [18]), and the Snaith-Hamilton Pleasure Scale (SHAPS; [19]), a measure of hedonic tone inversely related to anhedonia. Participants were compensated by 2 course credits. BDI scores ranged from 0 (i.e., non-depressed) to 54 (i.e., severely depressed range).

### Bandit task

Participants completed 30 bandit games of 16 trials each on a computer. On each trial, participants were allotted one token and had to choose from among 2 lottery arms in which to place the token. They then either received a 1-point reward (token turned green) or not (token turned red) from the chosen arm. After placing all 16 tokens, participants saw a brief screen with their total earned points in the game, and the next game began.

The reward probabilities (rates) were unknown to the participants except through experienced outcomes; they were told that these rates are redrawn and set at the beginning of each game. Thus, while our Bayesian learning model assumes that individuals believe that environmental statistics can undergo discrete, un-signaled changes without warning (see below), the reward rates were actually fixed (but unknown), and there were no actual change points in the present bandit task. Prior studies indeed suggest that individuals may still exhibit *sequential effects*, *a* persistent tendency to form expectations about upcoming stimuli based on *only* recent trials, which we have shown to be consistent with believing environmental statistics to undergo discrete change-points [12, 14, 20]. In practice, the reward rates for the 60 arms (2 for each of the 30 games) were pre-sampled and randomized in order for each participant. To generate the 60 reward rates, we sampled from a Beta distribution (α = β = 2), and then eliminated pairs that differed by less than 0.1 in probability. We found in preliminary simulation analyses that highly similar reward rates would lead to very noisy choice behavior that would be hard to model with the number of trials in the task.

**Fig 1:**
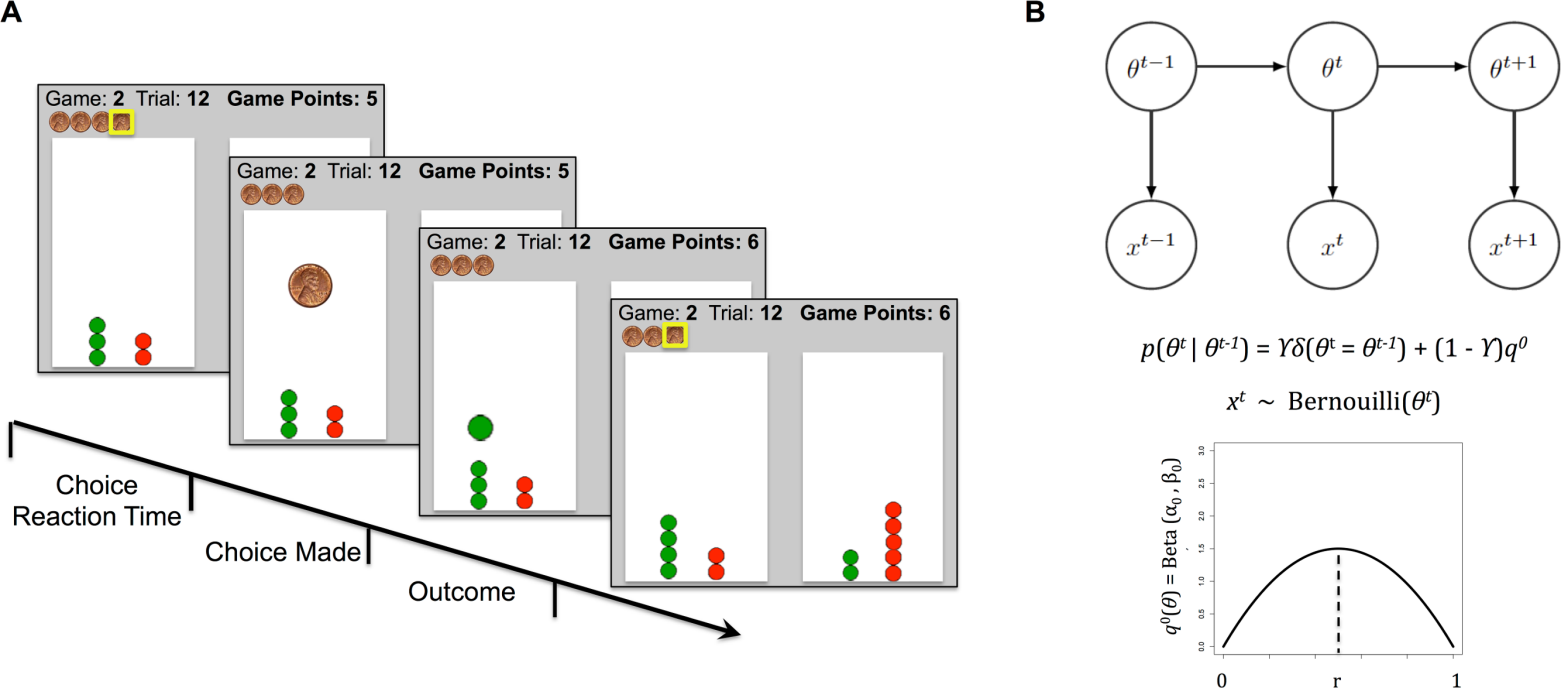
A. 2-arm bandit task trial timeline. Participants completed 30 games, each with 16 trials. On each trial of each game, participants had to assign one token (stacked horizontally at the top of the screen) to one of the two lottery arms. After placing each token, they either earned 1 point if the token turned green or zero points if the token turned red. Each trial lasted about 2s, including participants’ trial reaction time to assign a token and a 500ms outcome phase shoeing the token color once assigned. At the end of each 16-trials game, participants saw a brief screen (4s) with their total points earned in the game, and the next game followed. At the beginning of the task, participants were instructed to try earning as many points as possible in the task. They were further told upfront they would be paid in proportion to their total points earned in the game (actual paid amounts ranged from $5 to $10). Each trial decision and the arms reward rates were recorded. B. DBM illustration and the generative equations. The reward rate of each arm are assumed to be independently drawn at the start of a game from a Beta distribution *q*_0 =_ Beta (*α*_*0*_, *β*_*0*_), fixed throughout the game, and with mean *r = (α*_*0*_)/(*α*_*0*_ + *β*_*0*_)_._ DBM assumes that subjects believe that the reward rate *θ* for any arm can reset on any trial with probability 1-*γ*, otherwise it is the same value as the last trial.

Participants were instructed to try to maximize the points earned over all trials and all games. To additionally motivate the participants, we compensated them with a dollar amount proportional to their total points earned across all games at the end of the experiment (amounts paid ranged from $5 to $10; see Fig 1A for task details).

### Modeling

We modeled trial-by-trial learning using a form of the hidden Markov model, the Dynamic Belief Model (DBM), which assumes that the environmental statistics (i.e. reward rate for an arm in this task) undergo un-signaled changes [12–14]. We modeled the decision component using two decision policies: WSLS and Softmax.

#### Dynamic belief model

The model assumes that on each game, the two arms have reward rates, *θ*_*m*_, *m* = 1 or 2, each independently generated from the generic Beta prior distribution *q*^*0*^(*θ*_*m*_) = Beta (*α*_*0*_, *β*_*0*_) with mean *r = (α*_*0*_)/(*α*_*0*_ + *β*_*0*_). DBM further assumes that the reward probabilities for all arms can reset and be re-drawn from q^0^ on any trial with probability (1-*γ*), where *γ* is the *stability* parameter, which embodies the assumption of non-stationarity in the Dynamic Belief Model. Indeed, larger *γ* results in a more stable arm that changes reward rates less frequently (*γ* = 1 is a special-case arm that never changes reward rate at all). We therefore note that this modeling approach and the range of values we consider for the *γ* parameter (here .25 to 1) affords us the ability to capture both some level of sequential effects in individuals’ learning (when *γ* < 1) and also the possibility of fixed-belief updating and no sequential effects when *γ* = 1.

We use the notation *q*^*t*^_*m*_(*θ*^*t*^_*m*_): = *p*(*θ*^*t*^_*m*_|**x**^t^) to denote the posterior probability distribution over the reward rate for the *m*^th^ arm on the *t*^th^ trial, denoted *θ*^t^_m_, given the observed sequence of successes and failures from all previous trials, denoted ***x***^*t*^: = (*x*^*1*^*…x*^*t*^). At any trial, the subject’s prior belief has a Markovian dependence on last trial’s posterior:

$$p(\theta_{m}^{t}=\theta |x^{t-1})=\gamma q_{m}^{t-1}(\theta )+(1-\gamma )q^{0}(\theta )$$

To update the posterior after the current trial, for the chosen arm only (assuming it is the *m*^th^ arm), having observed the outcome *R*^*t*^_*m*_ (1 for a reward, 0 for no reward), the new posterior distribution for the chosen arm can be computed via Bayes’ rule:

$$q_{m}^{t}(\theta_{m}^{t})\propto P(R_{m}^{t}|\theta_{m}^{t})p(\theta_{m}^{t}|x^{t-1})$$

whereas the posterior for the un-chosen arm is the same as the prior at the beginning of the current trial (since there has been no new observation). We call the mean of the prior distribution, μ^t^_m_, the estimated reward rate for arm *m* (see Fig 1B). In the actual experimental design, the reward rates were fixed. This is one possible, special case setting also captured by the DBM, by assuming the probability of the reward rate changing on any trial is 0 (*γ* = 1), which we call the Fixed Belief Model [12, 14]. However, we have seen in many other experimental settings that subjects assume environmental statistics to be non-stationary even though they are truly fixed [12, 14, 21, 22].

#### Decision policies

We consider two models from the cognitive science and reinforcement learning literatures, previously shown to reliably capture human bandit choice [10, 23, 24]: the learning-independent heuristic *Win-stay*, *Lose-shift* (WSLS) and the more sophisticated learning-dependent *Softmax*. Those two models further provide complementary decision policies with respect to learning dependence and sophistication. Specifically, WSLS assumes that decision-maker chooses the same arm after obtaining a reward with probability *γ*^*w*^, but shifts away after a failure to reward with probability *γ*^*l*^ [9]. Thus, this model does not rely on any type of learning (e.g., expectations/beliefs about the unknown bandit arm reward rates), but is a heuristic model of its own. In contrast, the Softmax decision policy assumes that one chooses among the options with probabilities related to the inferred reward rates of the respective arms, but with a relationship that may depend on the individual [15]. Here, the choice probabilities are assumed to be normalized polynomial functions of the estimated reward rates, with polynomial parameter *b*, e.g. Pr(choosing arm 1) = *μ*_1_^*b*^/(*μ*_1_^*b*^ +*μ*_2_^*b*^), so that when *b* approaches infinity, the maximally rewarding option is always chosen (maximizing), when *b* is 1, it is matching [25], and when *b* is 0, the arms are chosen randomly (with equal probability). Thus, Softmax is a learning-dependent decision model, which in the present study relies on individuals’ estimated reward rates (*μ*_1_ and *μ*_2_) inferred with our Bayesian learning model DBM.

### Model fitting strategy

To capture individual differences in both learning and decision-making, we estimate for each individual his/her DBM stability parameter γ and decision policy parameters (*γ*^*w*^, *γ*^*l*^, *b*). However, we assume *α*_*0*_ *= β*_*0*_ *=* 2 in the prior distribution for DBM, instead of estimating them as free parameters, because simulations indicate that given our experimental design and sample size, we do not have sufficient statistical power to estimate *α*_*0*_ and *β*_*0*_ individually or even at the group level; on the other hand, assuming fixed *α*_*0*_ and *β*_*0*_, even if subjects actually have different *α*_*0*_ and *β*_*0*_, still yields relatively meaningful estimates of the other model parameters. The values of *α*_*0*_ *= β*_*0*_ *=* 2 were selected based the simulation results showing that such setting maximizes goodness of fit (i.e., R-squared) for linear regressions between inferred and true individual values of Softmax b parameters, as well as DBM γ. This is true in terms of peak R-squared value and maximizing R-squared for the widest range of individual parameter values, thus optimizing model fit for the majority of study participants (see S1 Text).

We estimate the overall likelihood of each model (WSLS or DBM+Softmax), as well as all model parameters for each model (fit on an individual subject basis), by maximizing the likelihood function (i.e., Maximum Likelihood Estimation, MLE). For each individual, we select the best model based on the minimum Bayesian Information Criteria (BIC), which is identical to MLE here, since DBM+Softmax and WSLS both have 2 free parameters. We also compare models using Bayes factor, defined as the ratio of model evidence (WSLS/Softmax), where model evidence is the marginal likelihood of the data given a model after integrating the uncertainty associated with the parameters of each model. Model fitting analyses and computations were performed using Matlab software (R2014 version) [26].

To assess the relationship between the affective measures (BDI, Anhedonia scale, STAI/State Anxiety) and each computational measure, we conduct five separate multiple linear regression analyses, with the following dependent variables, respectively: the model usage ratio and each model parameter (WSLS *γ*^*w*^, WSLS *γ*^*l*^, DBM *γ*, *Softmax b*). Predictors are entered in a hierarchical fashion, the first set of predictors including the 3 affective measures, and the second set including the interaction terms between the affective measures and model usage based on BIC selection (WSLS or Softmax). Such interactive patterns are of interest since a relationship between model parameter and affective measure may become manifest within a particular type of strategic use rather than across multiple strategic preferences. Regression analyses were performed using R statistical software (http://www.R-project.org [27]).

## Results

### Learning model

Similar to what we found in previous studies [14, 23, 28], participants had an average estimated stability parameter *γ* of .83, SD = .23, suggesting they behave as though they believe the environment is moderately stable, i.e., changing approximately once every 1/(1-*γ*) = 1/(1–0.83) = 5.9 trials. None of the affective measures were significantly related to *γ* (ps>.05).

### Decision policy

**Fig 2:**
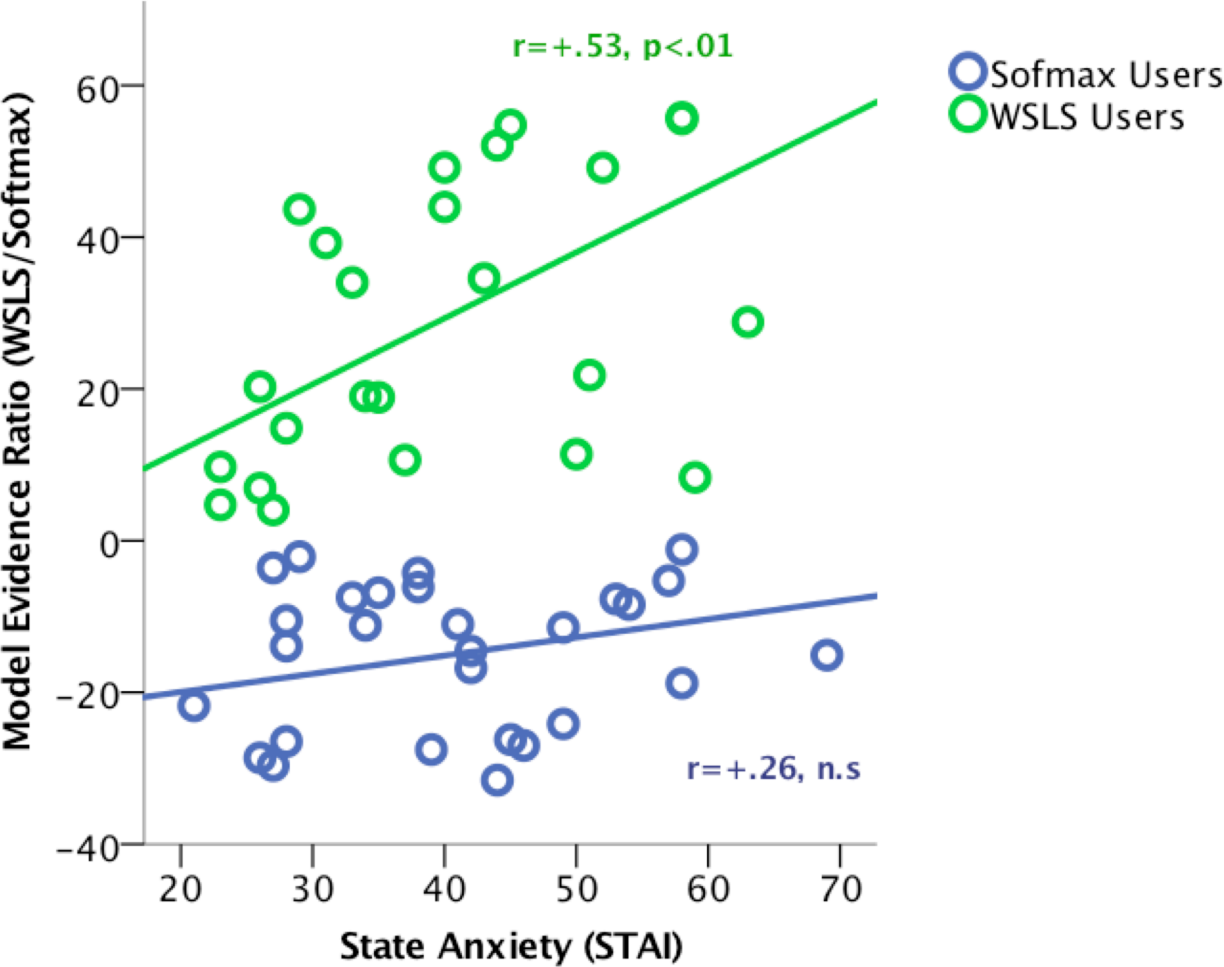
Correlation between state anxiety and model evidence ratio for WSLS relative to Softmax, by model usage group. WSLS = Win-Stay/Lose-Shift.

We found that 24 participants (45%) were best fit by WSLS, whereas 29 participants (55%) were better fit by a learning-dependent Softmax strategy. BIC and Bayes factor agreed on all cases (for model fit performance estimates based per-trial likelihood, see S2 Text). In terms of affective measures, there was a significant interaction between state anxiety and model usage type on the degree of model evidence (WSLS/Softmax; Stand. B = +.59; t = 2.9, p = .005; Full model R^2^ adjusted = .704). Specifically, a positive relationship between state anxiety and Bayes factor (WSLS/Softmax model evidence ratio) was observed in WSLS “users” (r = +.53, p = .008), but this relationship was not statistically significant in Softmax “users” (r = +.26, p = .158; see Fig 2). Other affective measures were not significantly related to the value of the Bayes Factor for an individual.

**Fig 3:**
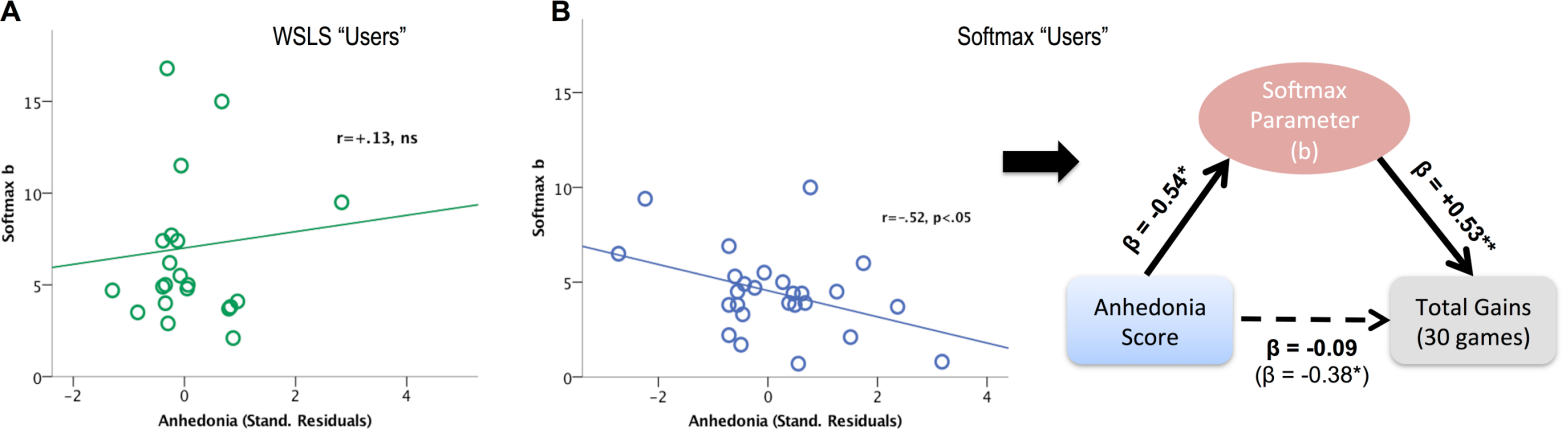
A. No significant correlation between Anhedonia and Softmax *b* parameter in WSLS “users”, r = +.13, ns. B. Left: Negative correlation between anhedonia and Softmax parameter in Softmax “users”, r = -.52, p < .05; Right: Mediation analysis with hierarchical linear regression models. Softmax b parameter was found to fully mediate the negative relationship between anhedonia and total points earned in the bandit task (among Softmax “users”, n = 29).

For individual decision policy parameters, we found a significant interaction between anhedonia (reverse-coded SHAPS score) and model usage type on the Softmax parameter (*b*; Stand. B = +2.08; t = 2.8, p = .007; Full model R^2^ adjusted = .292). Specifically, there was a negative relationship between anhedonia and Softmax *b* in Softmax “users” (r = -.52, p = .004), but no statistically significant relationship in WSLS “users” (r = +.13, p = .568; see Fig 3). We did not find any significant relationship between affective measures and WSLS parameters (*γ*^*l*^ or *γ*^*w*^).

### Relationship between model parameter and performance

Based on the above results in Softmax “users”, we investigated the relationship between the Softmax b parameter, anhedonia, and task performance (i.e., total points earned). Specifically, we assessed for any potential mediating role of the Softmax parameter in the relationship between anhedonia and task performance. Using hierarchical regression analyses to assess for mediation [29], we observed that a higher Softmax *b* parameter was associated with more total points earned (B = +.59,p = .006; R^2^ adjusted = .326). Another single predictor model showed that anhedonia was negatively related to total points earned (B = -.38,p = .04; R^2^ adjusted = .147). Importantly, adding *b* as a second predictor in this model removed the effect of anhedonia (B = -.09,p>.05), leaving *b* as the only significant predictor of total earned points (B = +.53, p = .01; R^2^ adjusted = .292), consistent with a full mediating role of this parameter in the negative impact of anhedonia on performance (see Fig 3B).

## Discussion

In this study, we applied two decision-making models, including a learning-independent strategy and a Bayesian learning dependent strategy, to human reward-based decisions in a bandit task, to quantify the relationship between affect attributes and cognitive learning and decision policy, in individuals with a range of depression severity. To model individuals’ beliefs updating, we used the Dynamic Belief Model (DBM), a Bayesian iterative inference model which assumes the environment to undergo unsignaled and discrete changes [14]. We note that the DBM formulation is related, though not mathematically equivalent, to a standard RL formulation [11, 30, 31], in that the stability parameter γ in the DBM controls the exponential forgetting (14), as does the learning parameter for incorporating the prediction error in a standard RL approach (e.g. Q learning). However, DBM allows the prior to repeatedly exert an influence, as opposed to only once at the beginning in typical model-free RL, and γ controls the relative importance of this continual prior influence. In addition, DBM includes the no-change case as a special case (stability parameter = 1), so it is possible for us to identify a subject as believing the reward rate to be fixed (and thus only incorporate the prior once at the beginning) on an individual basis, if appropriate. Decision-making was modeled with two well-established decision policies from the cognitive science and reinforcement learning literature: Win-stay/Lose-shift (WSLS) and Softmax. Interestingly, while the combination of DBM learning and Softmax strategy provided the best model fit for a majority of individuals (55%), a significant proportion of participants (45%) primarily relied on a WSLS strategy, a more learning-independent, heuristic model. This is consistent with a growing literature suggesting that WSLS behavior can sometimes prevail in exploratory reward-based decision tasks [32].

We found that, among individuals better fit with a Softmax policy, those reporting higher levels of anhedonia exhibited lower reward sensitivity, as reflected by lower values of the Softmax parameter (*b*). This parameter can be thought of as an index of the importance of reward in driving behavior. Specifically, when this parameter is large, the choice is driven more by the current mean estimates of reward rate on the options; however, when this parameter is small, we cannot tell whether an individual is simply being noisy or foregoing immediate reward in order to maximize expected future cumulative reward. The negative relationship between anhedonia and Softmax-based reward sensitivity is consistent with robust evidence of reduced reward seeking and approach behavior in depressive pathology, including for social cues, e.g., smiling human faces, [5, 33] and monetary rewards [3, 34]. A limitation of this finding is that Softmax does not explicitly assess the relative value of future exploratory gain versus immediate exploitative gain, but rather uses a single parameter to heuristically “loosen” up the choice policy relative to the estimated reward rates of the given options. Consequently, this policy model is insensitive to the number of trials left (i.e., horizon) relative to other models such as Knowledge Gradient or the optimal policy, which would require significantly larger datasets to do model selection and parameter estimation (12, 13). Thus, while our modeling framework does not tell us what precise factors (e.g. reward insensitivity, pure noise, or strategic maximization of longer-term reward attainment) drive high-anhedonia individuals to be more stochastic and explore “more”, what it does show is that high-anhedonia individuals give less direct weight to immediate reward in choice behavior. More generally, this finding corroborates a role of decreased hedonic tone in biasing the selection and execution of reward-based choice behavior [2, 3, 34, 35]. However, future studies are needed to tease apart the relationship between anhedonia and other aspect of reward sensitivity (e.g., sensitivity to short-term vs long-term rewards, including over time and practice with the task) or other factors (e.g., uncertainty, processing noise). For instance, while there is evidence of reduced neural representation of reward prediction errors in depression, a recent study suggests this may only be the case in a learning context, with no difference observed within non-exploratory task [36].

We also found that among those subjects whose choices are better captured by WSLS than Softmax, this tendency to use a learning-independent WSLS strategy was positively correlated with state anxiety. This relationship did not reach statistical significance in those primarily using a Softmax strategy. This interaction pattern and absence of significant correlation in the overall sample first suggests that anxiety may only relate weakly to strategic choice generally, and that such computational construct may be better explained by other factors not measured here (e.g., trait personality, genetics, etc). Secondly, within those individuals with a more marked tendency to use a WSLS strategy, this strategic preference appears exaggerated among those with higher state anxiety. In other words, state anxiety may only have a modulating effect on the degree of reliance on such learning-independent strategy, rather then a direct effect on selecting such strategy. Anxiety has more typically been associated with increased punishment sensitivity and negative expectations [8], rather than altered reward responsiveness. However, stress and trauma exposure have been linked to decreased reward responsiveness [4, 37], which may promote a less sophisticated learning-intensive strategy during reward-based exploratory decision-making. In addition, there is some evidence that physiological arousal, as observed in states of higher anxiety, may reduce cognitive resources and negatively impact executive control and goal-directed behavior [38], while promoting reliance on habitual/prepotent actions [39, 40]. Thus, one potential interpretation of our finding is that, higher anxiety may interfere with reward-seeking behavior by promoting a simpler, reactive strategy with less reliance on reward learning or prediction. Specifically, higher anxiety may be correlated with a greater tendency to ignore learned reward information when unexpectedly faced with the absence of reward). Another possible explanation for the observed relationship between state anxiety and WSLS reliance is increased punishment sensitivity in more anxious individuals. For instance, while the present paradigm does not explicitly include loss outcomes, the absence of reward when one is expecting can act as a negative/punishing outcome in a reward prediction error sense. We note, however, that we did not observe any significant relationship between the lose-shift (LS) parameter and affective measures, or between the LS parameter and the model evidence ratio, in the present study, which could be due to low statistical power rather than absence of such relationships. Future work, which explicitly includes gains and losses in a bandit paradigm, combined with a modeling approach capturing both gain and loss sensitivity/avoidance, for instance with distinct learning rates (see [41]), would allow us to test this hypothesis. In this respect, we surmise that an incremental learning model, such as a standard Q-learning RL approach, may be particularly useful in providing additional insights on the relationship between anxiety and learning horizon. However, we note that we did fit a standard RL model combined with a Softmax policy to the present data and, while we found it to be statistically indistinguishable from the DBM+Softmax model in terms of predicting subject choices (DBM: 69%, RL: 73%, p = 0.24), we were not able to replicate the observed relationship between anxiety and WSLS/Softmax strategic preference (nor did we observe any relationship between RL learning rate parameter and any of the other affective measures; see S3 Text). This could be due to DBM and RL not being quite the same model, since in simulations that match subjects’ actual experienced choices and outcomes, they only agreed with each other on 81% of the trials in terms of which arm is more rewarding. It would be a fruitful direction for future research to assess how RL and DBM are different and what type of experimental settings would best differentiate them, which is beyond the scope of this study.

Despite the suggestion of impaired reward learning in depression in the literature [2–4], we did not find any strong evidence of affective measures impacting Bayesian learning, at least in terms of the parameter reflecting the rate of change in the reward environment, or equivalently the effective memory window used to learn reward rates changes [12–14]. While this absence of significant finding could be due to lack of statistical power, one possibility is that our sample contained rather moderate severity of depression, while more severe depression and anhedonia could impact learning. Another possibility is that depression impacts learning not via expected rate of changing or the memory window, but rather via a baseline, pessimistic bias that reward rates are generally low in the environment–our experimental design and sample size did not allow us to assess this hypothesis directly. Both of these possibilities point to fruitful directions of future research.

Using a Bayesian learning-based modeling framework of decision-making, we found that anhedonia was associated with a lower reward-maximization bias, whereas state anxiety was associated with higher reliance on a learning-independent Win-Stay/Lose-Shift strategy. Adding to previous computational work linking depressed mood to altered exploratory decision-making [42], our results provide complementary evidence that anhedonia and anxiety symptoms in depression may distinctly alter reward-seeking strategy independently of reward learning. Interestingly, depression severity (as measured by the BDI) was not significantly related to any of the model parameters or policy usage measures. We suspect this may be due to the relatively heterogeneous nature of such measure, which encompasses multiple symptoms of depression (e.g., somatic, emotional, etc.) into one global indicator of clinical impairment, and therefore be more weakly associated with decision-making computational parameters, relative to more cognitively specific measures such as state anxiety and anhedonia.

In sum, by providing sophisticated representations of individuals’ internal beliefs and strategic computations, a computational approach can disentangle how various affective dimensions distinctly bias learning and decision-making. These subtle alterations can in turn inform the development of targeted behavioral interventions aiming to optimize decision-making in order to improve mood.

## Supporting information

S1 Text Model fitting and parameter setting strategy. (DOCX)

S2 Text Supplemental model fit comparison. (DOCX)

S3 Text Comparison of DBM and standard reinforcement (RL) models. (DOCX)

## References

[1] Eshel N, Roiser JP. Reward and punishment processing in depression. Biological psychiatry. 2010;68(2):118–24. doi: 10.1016/j.biopsych.2010.01.027 20303067 10.1016/j.biopsych.2010.01.027

[2] Gradin VB, Kumar P, Waiter G, Ahearn T, Stickle C, Milders M, et al Expected value and prediction error abnormalities in depression and schizophrenia. Brain. 2011;134(6):1751–64. 21482548 10.1093/brain/awr059

[3] Henriques J, Davidson RJ. Decreased responsiveness to reward in depression. Cognition & Emotion. 2000;14(5):711–24.

[4] Pizzagalli D, Iosifescu D, Hallett LA, Ratner KG, Fava M. Reduced hedonic capacity in major depressive disorder: evidence from a probabilistic reward task. Journal of psychiatric research. 2008;43(1):76–87. doi: 10.1016/j.jpsychires.2008.03.001 18433774 10.1016/j.jpsychires.2008.03.001 PMC2637997

[5] Heller AS, Johnstone T, Shackman AJ, Light SN, Peterson MJ, Kolden GG, et al Reduced capacity to sustain positive emotion in major depression reflects diminished maintenance of fronto-striatal brain activation. Proceedings of the National Academy of Sciences. 2009;106(52):22445–50. 10.1073/pnas.0910651106 PMC2796908 20080793

[6] Pizzagalli Holmes AJ, Dillon DG Goetz EL, Birk JL, Bogdan R, et al Reduced caudate and nucleus accumbens response to rewards in unmedicated individuals with major depressive disorder. American Journal of Psychiatry. 2009;43:76–87. 10.1176/appi.ajp.2008.08081201 PMC2735451 19411368

[7] Wang L, LaBar KS, McCarthy G. Mood alters amygdala activation to sad distractors during an attentional task. Biological psychiatry. 2006;60(10):1139–46. doi: 10.1016/j.biopsych.2006.01.021 16713587 10.1016/j.biopsych.2006.01.021

[8] Olvet DM, Hajcak G. The error-related negativity (ERN) and psychopathology: Toward an endophenotype. Clinical psychology review. 2008;28(8):1343–54. doi: 10.1016/j.cpr.2008.07.003 18694617 10.1016/j.cpr.2008.07.003 PMC2615243

[9] Robbins H. Some aspects of the sequential design of experiments. Bulletin of the American Mathematical Society. 1952;58:527–35.

[10] Daw ND, O'Doherty JP, Dayan P, Seymour B, Dolan RJ. Cortical substrates for exploratory decisions in humans. Nature. 2006;441(7095):876–9. doi: 10.1038/nature04766 16778890 10.1038/nature04766 PMC2635947

[11] Behrens TEJ, Woolrich MW, Walton ME, Rushworth MFS. Learning the value of information in an uncertain world. Nature neuroscience. 2007;10(9):1214–21. doi: 10.1038/nn1954 17676057 10.1038/nn1954

[12] Zhang S, Yu JA, editors. Forgetful Bayes and myopic planning: Human learning and decision-making in a bandit setting. Advances in neural information processing systems; 2013.

[13] Zhang S, Yu JA. Cheap but Clever: Human Active Learning in a Bandit Setting. Ratio. 2013;12(13):14.

[14] Yu A, Cohen J. Sequential effects: Superstition or rational behavior. Advances in neural information processing systems. 2009;21:1873–80. PMC4580342 26412953

[15] Luce R. Individual Choice Behavior. New York: Wiley; 1959.

[16] Doya K, Samejima K, Katagiri K-i, Kawato M. Multiple model-based reinforcement learning. Neural computation. 2002;14(6):1347–69. doi: 10.1162/089976602753712972 12020450 10.1162/089976602753712972

[17] Beck AT, Ward CH, Mendelson M, Mock J, Erbaugh J. An inventory for measuring depression. Archives of general psychiatry. 1961;4(6):561–71. 13688369 10.1001/archpsyc.1961.01710120031004

[18] Spielberger CD, Sydeman SJ. State-Trait Anxiety Inventory and State-Trait Anger Expression Inventory. 1994.

[19] Franken IHA, Rassin E, Muris P. The assessment of anhedonia in clinical and non-clinical populations: further validation of the Snaith–Hamilton Pleasure Scale (SHAPS). Journal of affective disorders. 2007;99(1):83–9. 16996138 10.1016/j.jad.2006.08.020

[20] Harlé KM, Shenoy P, Stewart JL, Tapert SF, Angela JY, Paulus MP. Altered Neural Processing of the Need to Stop in Young Adults at Risk for Stimulant Dependence. The Journal of neuroscience. 2014;34(13):4567–80. doi: 10.1523/JNEUROSCI.2297-13.2014 24672002 10.1523/JNEUROSCI.2297-13.2014 PMC3965782

[21] Ide JS, Shenoy P, Yu AJ, Li CS. Bayesian Prediction and Evaluation in the Anterior Cingulate Cortex Journal of Neuroscience. 2013;33(5): 2039–47. doi: 10.1523/JNEUROSCI.2201-12.2013 23365241 10.1523/JNEUROSCI.2201-12.2013 PMC3711643

[22] Yu AJ, Huang H. Maximizing masquerading as matching in human visual search choice behavior. Decision. 2014;1(4):275.

[23] Zhang S, Huang CH, Yu AJ, editors. Sequential effects: A Bayesian analysis of prior bias on reaction time and behavioral choice. Proceedings of the 36th Annual Conference of the Cognitive Science Society; 2014.

[24] Harlé KM, Zhang S, Schiff M, Mackey S, Paulus MP, Yu AJ. Altered statistical learning and decision-making in methamphetamine dependence: Evidence from a two-armed bandit task. Frontiers in psychology. 2015;6:1910 doi: 10.3389/fpsyg.2015.01910 26733906 10.3389/fpsyg.2015.01910 PMC4683191

[25] Herrnstein RJ, Laibson DI, Rachlin H. The matching law: Papers in psychology and economics: Harvard University Press; 2000.

[26] Matlab V. 8.3. 0.532 (R2014a). The MathWorks Inc, Natick, Massachusetts. 2014.

[27] Team RC. R: A language and environment for statistical computing R Foundation for Statistical Computing, Vienna, Austria 2013. 2014.

[28] Ma N, Yu AJ. Statistical Learning and Adaptive Decision-Making Underlie Human Response Time Variability in Inhibitory Control. Frontiers in psychology. 2015;6:1046 doi: 10.3389/fpsyg.2015.01046 26321966 10.3389/fpsyg.2015.01046 PMC4531239

[29] Baron RM, Kenny DA. The moderator–mediator variable distinction in social psychological research: Conceptual, strategic, and statistical considerations. Journal of personality and social psychology. 1986;51(6):1173 3806354 10.1037//0022-3514.51.6.1173

[30] Cohen JD, McClure SM, Angela JY. Should I stay or should I go? How the human brain manages the trade-off between exploitation and exploration. Philosophical Transactions of the Royal Society B: Biological Sciences. 2007;362(1481):933–42. 10.1098/rstb.2007.2098 PMC2430007 17395573

[31] Daw ND, Doya K. The computational neurobiology of learning and reward. Current opinion in neurobiology. 2006;16(2):199–204. doi: 10.1016/j.conb.2006.03.006 16563737 10.1016/j.conb.2006.03.006

[32] Worthy DA, Hawthorne MJ, Otto AR. Heterogeneity of strategy use in the Iowa gambling task: a comparison of win-stay/lose-shift and reinforcement learning models. Psychonomic bulletin & review. 2013;20(2):364–71. 23065763 10.3758/s13423-012-0324-9

[33] Dunn BD, Dalgleish T, Lawrence AD, Cusack R, Ogilvie AD. Categorical and dimensional reports of experienced affect to emotion-inducing pictures in depression. Journal of abnormal psychology. 2004;113(4):654 doi: 10.1037/0021-843X.113.4.654 15535797 10.1037/0021-843X.113.4.654

[34] Harlé K, Allen JJ, Sanfey AG. The impact of depression on social economic decision-making. Journal of Abnormal Psychology. 2010;119(2):440–7. doi: 10.1037/a0018612 20455617 10.1037/a0018612 PMC2869467

[35] Berridge KC, Robinson TE. What is the role of dopamine in reward: hedonic impact, reward learning, or incentive salience? Brain research reviews. 1998;28(3):309–69. 9858756 10.1016/s0165-0173(98)00019-8

[36] Rutledge RB, Moutoussis M, Smittenaar P, Zeidman P, Taylor T, Hrynkiewicz L, et al Association of Neural and Emotional Impacts of Reward Prediction Errors With Major Depression. JAMA psychiatry. 2017. 10.1001/jamapsychiatry.2017.1713 PMC5710549 28678984

[37] Nawijn L, van Zuiden M, Frijling JL, Koch SBJ, Veltman DJ, Olff M. Reward functioning in PTSD: A systematic review exploring the mechanisms underlying anhedonia. Neuroscience & Biobehavioral Reviews. 2015;51:189–204. 25639225 10.1016/j.neubiorev.2015.01.019

[38] Robbins T, Arnsten A. The neuropsychopharmacology of fronto-executive function: monoaminergic modulation. Annual review of neuroscience. 2009;32:267 doi: 10.1146/annurev.neuro.051508.135535 19555290 10.1146/annurev.neuro.051508.135535 PMC2863127

[39] Schwabe L, Wolf OT. Stress prompts habit behavior in humans. The Journal of neuroscience. 2009;29(22):7191–8. doi: 10.1523/JNEUROSCI.0979-09.2009 19494141 10.1523/JNEUROSCI.0979-09.2009 PMC6666491

[40] Dias-Ferreira E, Sousa JC, Melo I, Morgado P, Mesquita AR, Cerqueira JJ, et al Chronic stress causes frontostriatal reorganization and affects decision-making. Science. 2009;325(5940):621–5. doi: 10.1126/science.1171203 19644122 10.1126/science.1171203

[41] Frank MJ, Moustafa AA, Haughey HM, Curran T, Hutchison KE. Genetic triple dissociation reveals multiple roles for dopamine in reinforcement learning. Proceedings of the National Academy of Sciences. 2007;104(41):16311–6. 10.1073/pnas.0706111104 PMC2042203 17913879

[42] Blanco NJ, Otto AR, Maddox WT, Beevers CG, Love BC. The influence of depression symptoms on exploratory decision-making. Cognition. 2013;129(3):563–8. doi: 10.1016/j.cognition.2013.08.018 24055832 10.1016/j.cognition.2013.08.018 PMC3809321

