# Supplementary material for: Anhedonia and anxiety underlying depressive symptomatology have distinct effects on reward-based decision-making
Source: PLoS One. 2017 Oct 23;12(10):e0186473. doi: 10.1371/journal.pone.0186473 (PMC5653291; doi:10.1371/journal.pone.0186473)
Supplement: S1 Text — (DOCX) [file pone.0186473.s001.docx]

**S1 Text. Model fitting and parameter setting strategy**

As we had a relatively small amount of data per subject, we had to consider the trade-off between maximizing statistical power by reducing the number of parameters to be, and minimizing the bias associated with fixing certain parameters. The following discussion describes the simulations we performed to motivate our decision to fixed the DBM prior for all subjects to a Beta(2,2) distribution, i.e., *α_0_=β_0_=*2, which is consistent with a mean of .5 (the same as the actual mean reward probability in the experiment).

Simulations: We ran 20 simulations, with identical parameter settings and only differing in generative noise, to estimate how well the Softmax *b* and DBM *γ* parameters can be recovered, by computing the R-squared statistic between true and estimated parameter values. Each simulation consists of 52 simulated “subjects”, each with a randomly sampled DBM learning parameter (γ) between 0.25 and 1, and a Softmax parameter (b) between 0.5 and 12.5. We simulate 16 variants of each subject, each with a different setting of the Beta prior parameters alpha0 (1, 2, 3, or 4) and beta0 (1, 2, 3, or 4). We generate synthetic data for each rendition (setting of Beta prior) for each “subject”, by having each subject “play” 30 games of 16 trials (the same as the actual experiment), and where the reward rates of the arms in the 30 games are identical as those used in the experiment, though we cannot use the actual sequence of observations from the experiment, since our simulated subject will have a different sequence of choices; so we generate the reward one each chosen arm according to the underlying reward probability.

A “subject” updates its internal beliefs about the arms according to its DBM parameters and the observed rewards; it then makes a (stochastic) choice based on posterior means and the softmax parameter. We then use the same MLE algorithm used in the paper to estimate the Softmax and DBM parameters for each simulated subject. For each parameter estimation (Softmax and DBM alpha), three figures were drawn highlighting different comparisons. In each figure, the Y-axis represents the R-square statistics of the linear regression between estimated and the true individual parameter that is used in the simulation, while the X-axis represent the range of true individual parameter values.

Figures A (Softmax b) and B (DBM ) illustrate how different degrees of freedom in the assumption about the DBM prior affects the ability of the MLE algorithm to estimate the model parameters. In these figures, the alpha and beta parameters of the DBM prior are set as: a) free parameters, b) having a fixed sum of *α_0_+β_0_* =4, and c) Beta(2,2). These figures make apparent the superiority of Beta(2,2) relative to leaving those free or merely setting the sum at 4, in terms of achieving a significantly higher R-squared statistic, for a wide range of true individual parameter values.

Having determined that fixing both alpha0 and beta0 in the DBM prior is a superior approach given our data set size, we then investigate how different assumptions of these fixed values may affect the estimation performance. Figures C (Softmax *b*) and D (DBM *γ*) show that Beta(2,2) achieves comparable or better estimation performance than Beta(1,1), Beta(3,3), and Beta(4,4), for the full range of “true” Beta prior settings. Specifically, average R^2^ values for softmax parameter b for Beta(1,1), Beta(2,2), Beta(3,3), Beta(4,4) are 0.6281, 0.7007, 0.6895, and 0.6505, respectively (see Figure C). Average R^2^ values for the DBM gamma parameter for Beta(1,1), Beta(2,2), Beta(3,3), Beta(4,4) are .4964, 0.5192, 0.5175, 0.5097, respectively (see Figure D). Importantly, we note that the individual parameter values produced by these different settings are highly correlated (r>.95; R^2^ >.90) and thus, not surprisingly, produce the same results in terms of correlation with affective measures (both qualitatively and in terms of statistical significance).

**Fig A**

**Fig B**

**Fig D**

**Fig C**
