# Supplementary material for: Anhedonia and anxiety underlying depressive symptomatology have distinct effects on reward-based decision-making
Source: PLoS One. 2017 Oct 23;12(10):e0186473. doi: 10.1371/journal.pone.0186473 (PMC5653291; doi:10.1371/journal.pone.0186473)
Supplement: S2 Text — (DOCX) [file pone.0186473.s002.docx]

**S2 Text. Supplemental model fit comparison.**

To provide an additional estimate of model fit based on model/data agreement, the average per-trial likelihood (i.e., average probability of the model assigned to the bandit arm options chosen by participants) was computed for each model use subgroups (WSLS and DBM/Softmax users), which is presented in the graph below. For each model, average per-trial likelihood was significantly above the baseline chance level of 50% (ps<.001)
