# Supplementary material for: Anhedonia and anxiety underlying depressive symptomatology have distinct effects on reward-based decision-making
Source: PLoS One. 2017 Oct 23;12(10):e0186473. doi: 10.1371/journal.pone.0186473 (PMC5653291; doi:10.1371/journal.pone.0186473)
Supplement: S3 Text — (DOCX) [file pone.0186473.s003.docx]

**S3 Text:** Comparison of DBM and standard Reinforcement (RL) models:

We ran a standard Q-learning RL model (initial reward rate of .5 and learning rate value range of 0-1) combined with a Softmax strategy (same range of Softmax b values as for DBM-based learning) and investigated how it compared to our Softmax+DBM approach.

First, when we fit DBM+softmax and RL+softmax to subjects’ actual experienced choices and outcomes, we find that the prediction by DBM and RL in terms of which arm is more rewarding on each trial agree with the subjects’ actual choices in a statistically indistinguishable manner (DBM: 69%, RL: 73%, p=.24). However, this does not imply that DBM & RL are in general the same model, since they only agree with each other on 81% of the trials which arm is more rewarding (they disagree on 19% trials). This result implies that our experimental design and dataset size are not ideal for statistically distinguishing the two models from each other and measure their relative fits to human data. Future studies including more arms or looking at a broader array of reward environments would serve this purpose better, which was beyond the scope of the current study.

Second, in terms of the relationship between model parameters and affective measures, we observe that 1) the Softmax b parameters are highly correlated between DBM and RL approaches (r=.77, p<.001) and the anhedonia findings are replicated using the RL model (i.e., relationship between anhedonia and Softmax b is still present in Softmax users: r=-.45, p=.015, and the mediation analysis pathways for how this relationship impacts total points earned in the task remains the same and statistically significant; for instance, adding b as a second predictor of total points earned removes the effect of anhedonia, B=-.10,p>.05, leaving *b* as the only significant predictor of total earned points, B=+.48, p=.007; R^2^ adjusted =.29).

However, we find that, when using a RL+Softmax model (instead of DBM+Softmax), the relationship between the WSLS/Softmax model evidence ratio and anxiety, and specifically the interaction pattern between anxiety and strategic use type, is no longer present (interaction term in regression analysis is p=. 46, and the correlation between model evidence ratio and anxiety in WSLS users is r=.18, p=.39).

Also, we did not observe any correlation between this model evidence ratio and other affective measures (anhedonia, BDI). Overall, this suggests that the use of DBM with Softmax in our modeling approach allows us to capture a relationship between strategic preference and anxiety, which is not apparent with a standard RL approach. This may well be due to the fact that that DBM & RL do not agree on which arm is the better arm for 19% of the trials, and thus DBM is capturing something about human data that RL cannot.

Finally, we also note that we did not find any correlation between the RL learning rate parameter and any of the affective measures (ps>.3).

Overall, these additional analyses suggest that: 1) a standard RL approach does not lead to any additional finding in terms of the relationship between affective measures and decision policy and/or learning parameters in the present study (although we do recognize that this absence of finding may be due to statistical power limitations and the specifics of our task, including a focus on reward vs no-reward outcomes and 2 arms, which may not allow us to capture other important aspects of exploratory decision-making); and 2) our Bayesian learning model combined with Softmax policy allows us to capture a particular type of relationship between anxiety and strategic preference, which an RL-type model is less sensitive to.
